# Temporally consistent longitudinal brain tumor segmentation using a temporal spatial transformer network

**DOI:** 10.1038/s41598-026-53242-8

**Published:** 2026-05-19

**Authors:** Sandeep Kumar Mathivanan, Shamala K. Subramaniam, Dafik, Sunder R., Sangeetha S. K. B., Siva Shankar S.

**Affiliations:** 1 School of Computing Science and Engineering, Galgotias University, Greater Noida, Uttar Pradesh 203201 India; 2 Department of Communication Technology and Network, Faculty of Computer Science and Information Technology, Universiti Putra Malaysia (UPM), Serdang, Selangor 43400 Malaysia; 3 Department of Mathematics, Faculty of Mathematics and Natural Sciences, University of Jember, Jember, Indonesia; 4 Manipal Institute of Technology Bengaluru, Manipal Academy of Higher Education, Manipal, India; 5 Department of Computer Science and Engineering, KG Reddy College of Engineering and Technology, Hyderabad, Telangana 501504 India

**Keywords:** Tumor segmentation, Temporal attention, Longitudinal MRI, Transformer networks, Medical imaging, Deep learning, Neural networks, Cancer diagnosis, Cancer, Computational biology and bioinformatics, Mathematics and computing, Medical research, Oncology

## Abstract

Proper monitoring of tumor progression and evaluation of treatment responses highly depend on longitudinal brain tumor segmentation from MRI data. Current deep learning methodologies have mainly concentrated on analyzing single-time-point images, which restricts the ability to incorporate temporal dynamics during the segmentation process. The study proposes a new approach called Temporal-Spatial Transformer Network (TST-Net), which can be used for longitudinal brain tumor segmentation. The proposed methodology involves a temporal attention module to integrate the progression-aware information across temporally successive MRI images and a spatial attention module to improve tumor detection. Preprocessing of BraTS longitudinal MRI data and subsequent training of TST-Net on pre-aligned datasets were done in an end-to-end fashion. Evaluation was done by Dice similarity coefficients and compared with current methods. TST-Net proved to be more effective than previous solutions, obtaining 86.0%, 88.0%, and 91.0% Dice scores for improving tumor segmentation, tumor core, and overall tumor segmentation, respectively. This study reveals that incorporating the temporal dimension along with spatial attention leads to increased accuracy of brain tumor segmentation. Incorporating temporal information and applying spatial attention can effectively decrease inconsistencies between follow-up images and contribute to improved tumor area detection. TST-Net can serve as an important tool for clinical applications. TST-Net presents an efficient solution to brain tumor segmentation due to its ability to combine both spatial and temporal attention.

## Introduction

Identification, monitoring, and accurate characterization of brain tumors remain pertinent challenges in the field of neuro-oncology that have significant implications for therapeutic approaches and patient prognosis. Brain gliomas and other cancers pose additional complexities due to their heterogeneous response to therapy, spatial heterogeneity, and temporal growth. With its superior ability to delineate soft tissue structures and perform multi-parametric scans using techniques such as T1, T1 post-contrast (T1c), T2, and FLAIR, Magnetic Resonance Imaging (MRI) has become the benchmark technique for brain tumor imaging. Post-MRI volume processing requires expert radiologist interpretation, especially longitudinal scans taken during therapy stages, which accounts for heterogeneity.

By applying deep learning to achieve automatic segmentation, current works mainly rely on 3D data and ignore the dynamics of tumor characteristic changes which play an essential role in achieving tailor-made prognoses and personalized treatments. CNN-based architecture, including the classic U-Net and its variants, are widely adopted to learn space-wise structures and hierarchy within one-time frame. These models cannot incorporate time-wise interactions because they perform exceptionally well in static cases.

To model the interactions over time, methods relying on Recurrent Neural Network such as Long Short-Term Memory and Gated Recurrent Unit. LSTM and GRU are applied in various tasks such as time series prediction and video segmentation. Since they are sequential models, LSTMs are prone to gradient vanishing, lack of transparency, and inefficiency of learning long-term interactions from 4D MRIs (3D space + 1D time). This is mainly because they can’t be executed in parallel.

Medical data spatio-temporal modeling has handled by temporal convolutional networks and graph approaches. Graph Convolutional Networks (GCNs) and Spatio-Temporal GCNs (ST-GCNs) utilize node and edge representations to model the relationship between timepoints or anatomical features. These techniques heavily rely on learning adjacent matrices or pre-defined graph structures, which would not be able to adapt to patients with heterogeneous tumor shapes and growth patterns. Temporal convolutional techniques employ dilated convolutions to deal with long-range dependencies. However, they are usually less flexible and will not be able to fit longitudinally imaged data, which sampled, usually the standard in clinical settings where scans performed at unscheduled intervals on a case-by-case basis of patient states and treatment protocols.

Sequence modeling in Natural Language Processing (NLP) has recently transformed by attention-based models, especially Transformers, which are also quickly taking over the fields of computer vision and medical imaging. They applied to temporal and spatial contextual understanding tasks because they have a natural capability for modeling global dependencies using self-attention mechanisms. In many kinds of 2D and 3D vision tasks, Vision Transformers (ViT), Swin Transformers, and hybrid models combining CNN backbones with attention mechanisms have shown state-of-the-art performance. Their use in longitudinal medical imaging is still largely unexplored, though. Merging these mechanisms with the processing of multi-modal, high-resolution 4D MRI data remains a challenging task due to substantial memory constraints, limited availability of longitudinally annotated datasets, and the complexity of jointly modeling inter-modal interactions and temporal dependencies, despite recent efforts employing temporal attention mechanisms in video and echocardiography analysis.

Moreover, recent work disregards temporal consistency, a relevant factor in longitudinal tracking, in favor of being concerned only with terminal segmentation accuracy. Gross tumor growth or regression estimates induced by temporal inconsistencies across timepoints in anticipated tumor boundaries, as well as compromising model reliability. In treatment response monitoring, where precise tracking of tumor volumetric changes informs decision making about continuing, switching, or terminating treatment, this dimension is especially important. Therefore, even with the considerable advances in static brain tumor segmentation, the domain continues to lack strong solutions that can both convey the temporal dynamics and spatial structure of the tumors in an interpretable way within a therapeutic context.

The way current methods manage data imbalance and variability of longitudinal data is another significant flaw. Necrotic core, tumor enhancement, and edema are only a illustrations of present-day tumor sub-regions that may possess different dimensions and signal intensities at different modalities and timepoints. The paucity of labeled longitudinal scans combined with class-unbalanced representation tends to result in biased models that are unable to generalize. Performance undermined by intermodality and interpatient heterogeneity.These phenomena cannot be managed by traditional data augmentation strategies and fixed loss weighting strategies; instead, more responsive solutions like self-supervised learning, modality-aware fusion, and uncertainty estimation warranted.

Furthermore, performance on carefully curated datasets is not enough to reach clinical deployment of deep learning models. Interpretability, generalizability, and real-time inference capabilities are critical for practical deployment. Although they are accurate, most current models either lack architectural transparency required for therapeutic trust or infer unrealistically slowly. Longitudinally stable, computationally efficient, accurate models that adapt with ease to new imaging protocols and domains with minimal retraining required to narrow the gap between experimental achievement and clinical feasibility. This two-dimensional deficiency in current research and practice is the motivation for this study.

Emerging segmentation models that account for the dynamic changes of tumor regions in a spatial sense over time takes advantage of the complementing nature of multi-modal MRI scans and gives temporally coherent outputs in an interpretable manner. This opens a chance to explore different architectures suitable for analyzing 4D tumors due to the lack of consistency seen in current temporal models for neuro-oncology and with the increase in available longitudinal datasets such as BraTS Longitudinal and IXI. The advancements in Transformer-based approaches and cross-domain generalization for developing efficient models across both the temporal and spatial domains are also to be considered.

Even though much success has been achieved in 3D tumor segmentation, it is apparent that the current methods do not adequately incorporate the dynamic nature of the tumor which is key for the prolonged monitoring of the disease. While CNN-based methods are inherently static, RNN and GCN models have largely failed due to the limitations of their design. Current efforts in terms of temporal modeling for multi-modal 4D medical imaging are not scalable, resilient, or coherent in a temporal sense. This gap in the field can be seen through the inability to develop clinically viable tools for tracking the tumors dynamically.

The main contributions are.

1. To design a novel Temporal–Spatial Transformer Network (TST-Net) integrating convolutional feature extraction with transformer-based temporal modeling to enable accurate 4D brain tumor segmentation across multiple longitudinal MRI timepoints.
2. To propose temporal attention mechanisms and self-supervised pretraining incorporated to improve segmentation accuracy, mitigate data imbalance, and ensure temporal consistency in tumor progression tracking.
3. To evaluate on the BraTS longitudinal dataset demonstrates that the proposed TST-Net outperforms state-of-the-art methods in both segmentation accuracy and tumor volume prediction, validating its effectiveness for longitudinal tumor analysis.

##  Related study

One of the essential elements in neuro-oncology today includes the accurate distinction of brain tumors through clinical imaging, offering significant data in diagnosis, surgery and reaction to therapy. Due to the better soft-tissue distinction offered by MRI, it has become the default method in this process. The manual segmentation of the tumor boundary proves to be a very time-consuming, subjective and difficult activity, leading to differences among experts. Thus, it accelerated the research and implementation of automatic algorithms, especially deep learning-based approaches that mimic and in some cases surpass the level of expertise of a human observer^1,2^. From simple CNNs to better neural networks that apply attention mechanisms and transformer architecture to take into account the complex nature of brain tumors.

CNNs were initially the leaders of the deep learning revolution for medical image segmentation, and the U-Net model and its variants dominated as a mainstream paradigm^3^. To obtain both local fine-grained knowledge and global contextual knowledge in a 2D or 3D image region effectively, the models make use of an encoder-decoder architecture with skip connections. This method implemented and utilized for brain tumor segmentation in studies. As evidenced by models such as ResUNet50^4^, progress has achieved with the use of residual connections to create deeper networks that are resistant to vanishing gradients. Other improvements are the addition of multi-scale feature extraction and attention, which allows the model to deal with larger areas of the image, hence making it more accurate and robust in segmentation^3^.

Lightweight separable spatial convolutional networks have put forward as an approach to developing more capable models that applied for use in clinical settings with modest processing capacity^5^. Another method called MAEU-NET offers innovative supervised architecture that is designed to improve segmentation performance^6^. On top of improving segmentation results’ quality and reality, Generative Adversarial Networks (GANs) have also used, with a double-scale multi-modality perceptual approach^7^. Architectures such as the 3D guided attention inception residual U-Net (GAIR-U-Net), that efficiently integrate information across various sequences with parallel feature aggregation, have proposed to overcome the problem of tumor segmentation from various MRI modalities^8–10^.

With these developments in place, long-range spatial relations in the image at times prove difficult to describe using these conventional CNN-based approaches. This has opened the door for novel architectural investigations. Transformer designs originally evolved for NLP but have also effectively used for deployment in computer vision and medical imaging. Instantaneous resolution of one of the biggest limitations of CNNs provided by its top self-attention mechanism, which is extremely effective in mimicking global context and long-range dependencies. Therefore, strong brain tumor segmentation models have emerged. Of greatest demand are hybrid architectures that leverage the contextual modeling strength of Transformers with the spatial feature extraction strength of CNNs.

Very accurate segmentation of sub-tumoral regions such as the enhancing tumor, necrotic core, and peritumoral edema is achieved by designs such as TransBTS, DE-UFormer, and 3DUV-NetR+, that tend to use a CNN-based encoder to get hierarchical feature maps and a Transformer-based module to understand the relationship between them^11–13^. SwinBTS is also an innovation that uses the Swin Transformer architecture to represent hierarchical context and achieve computational efficiency^14^. To further optimize the hybrid CNN-Transformer architecture, the TransSea model includes a semantic awareness module^15^. Through the development of systems that perform better than their respective CNN or Transformer-only counterparts, such models illustrate the value of synergy.

The designs like the shape-scale co-awareness network, where extra care taken in collecting tumor morphology on diverse scales to perform better 3D segmentation, are an example of such advancements achieved^16^. Another new horizon created by advancements in large-scale pre-trained medical base models, which pilot studies show could help in brain tumor analysis and diagnosis with little task-specific fine-tuning^17^. The use of multimodal MRI sequences such as T1-weighted (T1), post-contrast T1-weighted (T1c), T2-weighted (T2), and Fluid-Attenuated Inversion Recovery (FLAIR) images are crucial for clinical brain tumor imaging. Each sequence provides distinct information regarding various tissue properties as well as disease characteristics.

Segmentation must be precise depending on the successful fusion of modalities. Advanced models, for instance, TransBTS and SwinBTS, built natively multimodal for input and use different strategies to combine feature channels early or late in the network^13,14^. Because not all sequences contribute equally to the final prediction, research has also attempted the optimal acquisition sequence to obtain maximum information gain for AI-augmented segmentation. This has deep implications for clinical imaging protocol simplification^1^. These multimodal inputs used for more comprehensive studies than segmentation alone, such as lifetime prediction and tumor grading, where machine learning models learn to correlate imaging features with clinical outcomes^18^.

While few approaches utilize advanced techniques such as artificial bee colony optimization with fuzzy logic to delineate the tumor boundaries from multimodal data^19,20^, other systems have designed that utilize sequential progressive segmentation across modalities to robustly detect tumor regions^21^. The analysis of tumor behavior over time remains an innovative area in neuro-oncology, although much progress has achieved with segmenting static 3D brain volumes. Brain tumors are dynamic structures that develop, change, and react to therapy. This requires analysis of 4D data, including the temporal axis and the three geographic axes. Longitudinal study required tumor growth prediction, assessing the response to therapy, and unraveling the biology of tumor development^20,22^.

However, there are certain challenges in simulating this fourth dimension. The early studies in this space have used spatio-temporal convolutional LSTMs to learn from 4D longitudinal patient data for tumor growth prediction^22^ and extended segmentation networks to 4D CT volumes for brain tissue segmentation^10,23^. Studies have targeted the use of 4D flow MRI to investigate hemodynamics^24^ or fetal brain mapping development by producing 4D atlases of automated segmentations^25^. Despite this, most current methods process each time point individually, not necessarily accounting for the intricate, non-linear tumor growth processes or temporally coherent processes. This gap underscores the strong necessity of novel models tailored to the analysis of 4D spatiotemporal data.

With continued efforts in developing AI-powered neurosurgery platforms and interpretable models that are trusted and valued by clinicians, the final goal is to integrate such advanced tools into clinical practice^26,27^. These guidelines summarized in a full review of the topic between 2019 and 2023, along with significant accomplishments and uncovering the long-term limitations that promote research^2^. For brain tumor care, the overall trend is more dynamic, integrated, and therapeutically meaningful AI systems^28^. Key trends in automatic brain tumor analysis are uncovered by literature. In the first place, there is a discernible trend towards design from models that are exclusively CNN-based, like U-Net and its extensions^3,4,6,9^, to hybrid designs blending CNNs and Transformers for improved global context understanding^11–15^ and strong spatial feature extraction, respectively.

Multimodal MRI data is increasingly becoming the norm, and investigations are centering on advanced fusion techniques to leverage the complementary information from FLAIR, T1, T1c, and T2 sequences^1,12,14^. The studies are discussing beyond the basics to address tougher requirements, which include tumor grade assessment^29^, longevity prediction^18^, and understanding the tumor microenvironment^20^. Studies are beginning to study 4D datasets to study brain development and tumor growth over time, thereby shifting from static to dynamic image analysis, signaling the emergence of a new trend in the field of medical imaging^22,23,25^. The fact that most recently developed algorithms are geared for static 3D volumes and are applied to longitudinal data independently is a major setback.

This commonly resulted in segmentations that are not temporally coherent and are not informative about tumor dynamics because it neglects important temporal dependencies and relationships. The recurrent structure utilized by models that attempt spatio-temporal analysis, such as LSTMs^22^, can be even worse at recognizing intricate, long-range temporal structures than Transformers. Additionally, models are computationally demanding, making it difficult to integrate them into clinical workflows in real-time^5^. Class imbalance and sparsity of data continue to be issues, especially for tumor subtypes or sub-regions that occur infrequently and thus may need large human-annotated training datasets^17^. Most deep learning models are “black boxes” and do not have the interpretability required to be broadly trusted and accepted by clinicians^26^.

The current study on a Temporal-Spatial Transformer Network (TST-Net) for 4D brain tumor segmentation motivated by limitations of current methods. The main motivation is the necessity for a model that processes the temporal dimension in longitudinal MRI scans in an efficient and clear-cut way. The target TST-Net is to overcome the weakness of models with weaker recurrent networks or time points processed separately by adding a Transformer-based temporal attention mechanism^22^. This would help in developing tumor evolution models directly, optimizing temporal consistency in segmentation across time and improving growth prediction accuracy. The success of such neural network structures^12–14^ has prompted the design of the CNN-Transformer hybrid model, making sure the neural network utilizes both strong spatial feature extraction from local regions and global spatiotemporal modeling of features.

Self-supervised learning aims at acquiring rich feature representation in the absence of labeled data before training the network, and this is a response to the problem of sparse datasets. The TST-Net seems to fill the gap in existing studies and move the research from a static 3D framework to a dynamic 4D approach of understanding brain tumor growth.

## System methodology

### Dataset description

This study employs two publicly available magnetic resonance imaging (MRI) datasets-BraTS Longitudinal and IXI.

#### BraTS longitudinal dataset (primary supervised dataset)

The main dataset utilized for supervised training, validation, and assessment of the suggested Temporal-Spatial Transformer Network (TST-Net) is the Brain Tumor Segmentation (BraTS) Longitudinal dataset. The dataset made up multi-modal, multi-institutional MRI images obtained from patients with lower-grade glioma (LGG) and glioblastoma (GBM). The analysis of tumor growth, therapy response, and progression dynamics made it possible by the longitudinal MRI scans obtained at various clinical timepoints for each participant. Four common MRI modes offered for each scan T1-weighted (T1), post-contrast T1-weighted (T1c), T2-weighted (T2), Fluid-Attenuated Inversion Recovery (FLAIR).

The dataset curator’s skull-strip, co-register, and resample each scan to a common resolution. BraTS provides expert-annotated voxel-wise ground truth labels that defined by skilled neuroradiologists and verified by consensus. Clinically significant tumor sub-regions defined by these annotations Enhancing Tumor (ET), Tumor Core (TC), Edema (ED), Necrotic and Non-enhancing Tumor Core (NC). These labels allow for quantitative investigation of volumetric changes over time and accurate supervised learning for tumor sub-region segmentation. The dataset’s longitudinal nature makes it possible to describe each patient’s tumor as a 4D sequence (3D space + time), which is crucial for modeling tumor growth trajectories, analyzing treatment-induced morphological alterations, and measuring temporal consistency. The BraTS longitudinal dataset is the sole source of computation for all segmentation performance measurements, temporal consistency analysis, ablation experiments, and comparative assessments presented in this work. We follow the BraTS definition where TC includes enhancing and necrotic core regions.

#### IXI dataset (unsupervised pretraining dataset)

The IXI dataset, which made public by Imperial College London, made up only of MRI scans from healthy individuals that obtained using various scanners and acquisition techniques at three separate institutions. IXI lacks lesion annotations, tumor images, and pathological cases. The IXI dataset not used in this study for testing, validation, or training tumor segmentation. It used for anatomical representation learning and self-supervised pretraining. Before exposed to diseased data, the model can develop dependable, modality-invariant representations of normal brain anatomy, scanner variability, and intensity distributions across MRI modalities thanks to the integration of IXI.

For learning low-level and mid-level anatomical aspects including cortical structure, tissue borders, and modality-specific contrast patterns, the IXI dataset provides the same core MRI modalities (T1, T2, and occasionally FLAIR). When fine-tuning on the smaller, fully annotated BraTS dataset, this pretraining stage helps stabilize optimization, enhance convergence, and lessens overfitting. This pretrain–fine-tune paradigm is well-established in medical image analysis and is especially useful for transformer-based architectures that need strong initialization. It involves using large-scale healthy MRI data for unsupervised or self-supervised representation learning followed by supervised training on disease-specific datasets.

The objectives of TST-Net are supported by the combination of IXI and BraTS. While training on BraTS ensures that the model learns tumor-related spatiotemporal patterns precisely, pre-training on IXI ensures that the model learns anatomically and modality-specific features consistently. For longitudinal tumor segmentation, such an arrangement enhances robustness, generalizability, and consistency without compromising methodology.

### Preprocessing

Different modalities of MRI images using varied types of scanning devices and different resolutions constitute BraTS Longitudinal and IXI datasets. Prior to training the models, only one pre-processing pipeline was followed to ensure homogeneity in datasets and modalities. No knowledge about any tumor is required while performing pre-processing. All pre-processing is independent of modalities and datasets. To facilitate batch-wise learning and reduce the overhead, all MRI images are scaled to a constant three-dimensional image volume with constant resolution. While maintaining anatomical structures, this stage normalizes spatial dimensions across people and mediums.

Voxel-wise intensity normalization applied separately to each modality to address intensity variability across scanners and modalities. Z-score normalization conducted as follows:

$${\mathrm{I}}_{{{\mathrm{norm}}}} = {\mathrm{I}}_{{{\mathrm{raw}}}} - \mu /\sigma$$

where I_raw_​ denotes the raw voxel intensity, and µ and σ represent the mean and standard deviation of intensities within the corresponding volume. This normalization ensures that no single modality dominates the learning process due to scale differences.

To preserve consistent input dimensions and eliminate non-informative background areas, volumes center-cropped and padded to a standard size. This procedure ensures that both healthy (IXI) and diseased (BraTS) scans receive the same treatment because it is solely spatial and does not depend on tumor location or annotations. Gaussian filtering used to reduce high-frequency noise that could impede feature learning and enhance data quality. Intensity inhomogeneities resulting from magnetic field non-uniformities mitigated by using N4 bias field correction. These adjustments make it possible to extract features consistently and reliably throughout the whole brain volume.

#### Multi-modal alignment

To guarantee voxel-wise correspondence between modalities, all modalities (T1, T1c, T2, and FLAIR) are co-registered to a shared anatomical space. Effective multi-modal fusion in later network phases depends on this alignment.IXI scans used for self-supervised pretraining following preprocessing; tumor labels, masks, or region-specific supervision not used. For supervised fine-tuning and evaluation, preprocessed BraTS scans utilized. Expert-provided voxel-wise tumor annotations allow for the learning of tumor sub-regions and temporal progression. This preprocessing pipeline guarantees consistent, high-quality inputs while tightly limiting any leakage of tumor-specific information from healthy data.

### Algorithm for temporal-spatial transformer network (TST-Net)

**Fig. 1:**
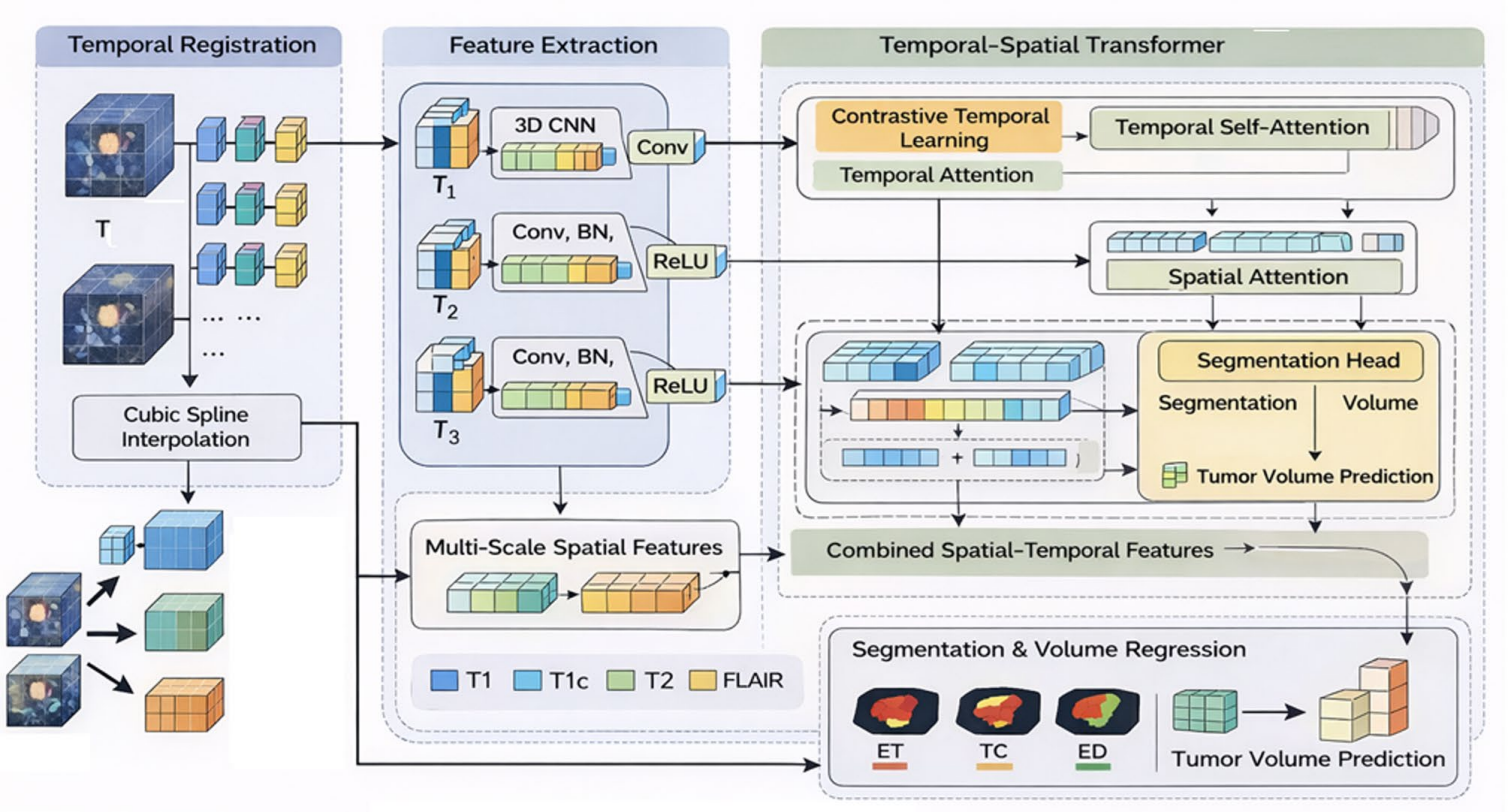
Proposed framework.

The model is pretrained in a self-supervised manner using IXI data without any tumor labels and subsequently fine-tuned using BraTS longitudinal data with full supervision. Using multi-modal 3D MRI data, the TST-Net (Temporal-Spatial Transformer Network) approach seeks to solve brain tumor segmentation and growth prediction in the future. For ensuring constant time intervals between images of various patients, the approach first begins with temporal registration of MRI data using cubic spline interpolation. Multi-scale feature extraction is subsequently done using a 3D Convolutional Neural Network (CNN) to capture spatial information in all the modalities (T1, T1c, T2, FLAIR). Contrastive Temporal Feature Learning is performed to enhance temporally consistency through temporally connecting neighboring time points to provide temporal smoothing for tumor progression. Figure 1 demonstrates the overall structure of the suggested framework. The workflow starts with temporal alignment of longitudinally acquired MRI images, followed by multiscale feature learning via a three-dimensional convolutional network. The obtained features fed into the Temporal-Spatial Transformer to learn temporal correlations among the time points and spatial associations within the scans by applying the attention mechanism. The model produces tumor segmentations and associated volumes.

**Step 1** The temporal alignment of multi-modal MRI data, where each sample $\:{X}_{MRI}\in\:{R}^{H\times\:W\times\:D\times\:4}$ consists of four modalities (T1, T1c, T2, FLAIR). H, W, and D stand for height, width, and depth respectively. It is very important for the time aspect T, since it is essential for studying tumor development. For harmonization of scans from different time intervals, cubic spline interpolation has been employed to modify the time intervals T_time_ of each scan in 3D MRI. Even though interpolation was done for normalizing the time intervals, it did not change the original order of acquisition of scans.

$$\:{T}_{aligned}=SplineInterp({X}_{MRI},{T}_{time})$$

This preprocessing step ensures that irregularities due to different scan acquisition times resolved and each timepoint is equally spaced, enabling a uniform analysis of tumor growth dynamics.

**Step 2** The multi-modal MRI scan at each timepoint passed through a 3D Convolutional Neural Network (CNN) to extract hierarchical spatial features. The 3D CNN architecture captures both low-level features (edges and textures) and high-level features (tumor boundaries). For a scan $\:{X}_{i}\in\:{R}^{H\times\:W\times\:D\times\:4}$, the network applies convolutional filters over all modalities simultaneously.

$${F}_{modality}=Conv3D\left({X}_{i}\right)$$

where the 3D feature map $\:{F}_{modality}$ constructed. This ensures the preservation of spatial relations within each modality, crucial for identifying fine-grained tumor regions. The learned features at each level of the network are crucial for later temporal and spatial feature fusion.

The convolutional backbones of the proposed architecture comprise a multi-scale three-dimensional encoder processing multi-modal MRI volume. The architecture comprises convolutional blocks with batch normalization and non-linear activation followed by down sampling blocks to extract hierarchical spatial features. Shallow convolutional blocks learn low-level features like texture and edges, whereas deep blocks learn high-level features like the boundary of tumors and structural context. Skip connections are used to maintain spatial resolution and enhance feature flow.

**Step 3** The data is longitudinally collected; the contrastive learning strategy has been used to improve temporal continuity. The objective is to make sure that the network learn a continuous representation of the tumor’s development through time. The process involves comparing the latent representations of z_t_ and z_t+1_ of patient p.

$$\:{L}_{contrastive}=-log\left(\frac{{e}^{sim({z}_{t},{z}_{i+1})}}{{\sum\:}_{i}^{\:}e{\:}^{sim({z}_{t},{z}_{i+1})}}\right)$$

where $\:sim({z}_{t},{z}_{i})$ represents the cosine similarity of the encoded features $\:{z}_{t}$ and $\:{z}_{i}$. This contrastive loss ensures that temporally adjacent features embedded closely in the latent space, enabling the model to track tumor progression effectively.

Temporal modeling is carried out via chronological ordering of MRI scans in individual timeline. All the scans at various timepoints considered sequential data points, and the features of all the scans combined into a temporal sequence. Unlike explicit encoding of positional information, the chronological information of each scan was kept by ordering sequences. The transformer learned correlations between scans at early timepoints and later timepoints, allowing the model to detect tumor evolution and growth pattern.

**Step 4** Incorporating spatial-temporal attention enhances the model’s ability to focus on both spatially important tumor regions and temporally relevant timepoints. For spatial attention, let $\:{F}_{spatial}\in\:{R}^{H\times\:W\times\:D}$ be the 3D feature extracted from each modality, and the spatial attention map $\:{A}_{s}$ computed as.

$$\:{A}_{s}=Softmax\left(Attention\right({F}_{spatial}\left)\right)$$

Similarly, a temporal attention map $\:{A}_{t}$ is learned by aggregating temporal features across timepoints.

$$\:{A}_{t}=Softmax\left(Attention\right({F}_{temporal}\left)\right)$$

These attention maps fused together in the following manner to generate a spatial-temporal feature map.

$$\:{F}_{spatial-temporal}={A}_{s}\odot\:({A}_{t}\cdot\:{F}_{modality})$$

where $\:\odot\:$ denotes element-wise multiplication. This fusion helps the model concentrate on the most critical regions and time points, improving its capability to identify tumor growth and shrinkage over time.

**Step 5** A multi-head self-attention mechanism forms the core of the spatial-temporal transformer block. The feature map $\:{F}_{spatial-temporal}$ passed through a multi-head attention mechanism that teaches global dependencies between spatial and temporal features.

$$\:Z=MHSA\left({F}_{spatial-temporal}\right)$$

The result $\:Z$ contains refined spatial-temporal features that provide a deeper understanding of the tumor’s evolution. This allows the model to selectively focus on tumor regions that change over time, which is key for accurate segmentation and growth prediction.

Transformer module works with the features extracted and consists of several attention layers stacked together. Every attention layer consists of multi-head self-attention and feed-forward neural networks that are normalized. The use of attention in this module helps the model learn from dependencies from various time points, while parallel computation makes the process much faster than in recurrent networks. Residual connections were used to make training more stable.

**Step 6** The proposed model uses multi-task learning to predict both tumor segmentation and tumor volume. The segmentation loss $\:{L}_{seg}$ computed using binary cross-entropy. Binary cross-entropy combined with Dice loss to address class imbalance.

$$L_{{seg}} = - \sum\limits_{{c\epsilon \{ ET,TC,ED,NC\} }} {y_{c} {\mathrm{log}}(\widehat{{y_{c} )}}}$$

where $\widehat{{y_{c} }}$ represents the predicted probability for each class (Enhancing Tumor, Tumor Core, Edema, Necrotic Core), and $\:{y}_{c}$ are the ground truth labels. At the same time, the model performs regression to predict the tumor volume $\:V$ at each timepoint, using mean squared error (MSE)

$$L_{{volume}} = \frac{1}{N}\sum \: _{{i = 1}}^{N} (v\,_{{\hat{i}}} - v_{i} )^{2}$$

**Step 7** To predict the tumor’s growth over time, we use an exponential growth model that accounts for changes in tumor size over multiple timepoints. Given the tumor volume $\:{V}_{t}$ at time $\:t$, the predicted volume at the next timepoint $\:t+\varDelta\:t$ given by.

$$\:{V}_{t+\varDelta\:t}={V}_{t}\cdot\:exp\left(r\varDelta\:t\right)$$

where $\:r$ represents the growth rate of the tumor. The growth rate $\:r$ predicted by the model and optimized through regression.

The tumor volume prediction task incorporated as a complementary objective to enforce temporal consistency and enhance feature representation learning. By jointly optimizing segmentation and volume estimation, the model captures both spatial boundaries and global tumor progression trends, improving robustness in longitudinal analysis.

**Step 8** The total loss function $\:{L}_{total}$ combines the segmentation and volume prediction losses using weighting parameters $\:{\lambda\:}_{1}$ and$\:{\lambda\:}_{2}$.

$$\:{L}_{total}={\lambda\:}_{1}{L}_{seg}+{\lambda\:}_{2}{L}_{volume}$$

This ensures that the model learns to optimize both tasks simultaneously, balancing tumor segmentation accuracy with tumor volume prediction.

**Step 9** The model parameters $\:\theta\:$ optimized using the Adam optimizer with a learning rate$\:\eta\:$.

$$\:{\theta\:}_{t+1}={\theta\:}_{t}-\eta\:{\nabla\:}_{\theta\:}{L}_{total}$$

where $\:{\nabla\:}_{\theta\:}{L}_{total}$ is the gradient of the total loss with respect to the model parameters. Additionally, learning rate scheduling is employed to adaptively adjust the learning rate during training.

**Step 10** After obtaining the predicted segmentation mask $S^{ \wedge }$morphological operations applied to reduce noise and refine the mask boundaries.

$$S^{ \wedge } _{{refined}} = Morphological\;Processing\left( {S^{ \wedge } } \right)$$

This post-processing step helps smooth the tumor boundaries, ensuring more accurate segmentation results.

**Step 11** By applying the tumor growth model learned during training, the model predicts future tumor volumes at subsequent time points. This enables long-term tumor progression analysis, assisting clinicians in forecasting tumor behavior and guiding treatment decisions.

$$\:{V}_{future}={\sum\:}_{t}^{\:}\:{v}_{t}.exp\left({r}_{t}\varDelta\:t\right)$$

**Fig. 2:**
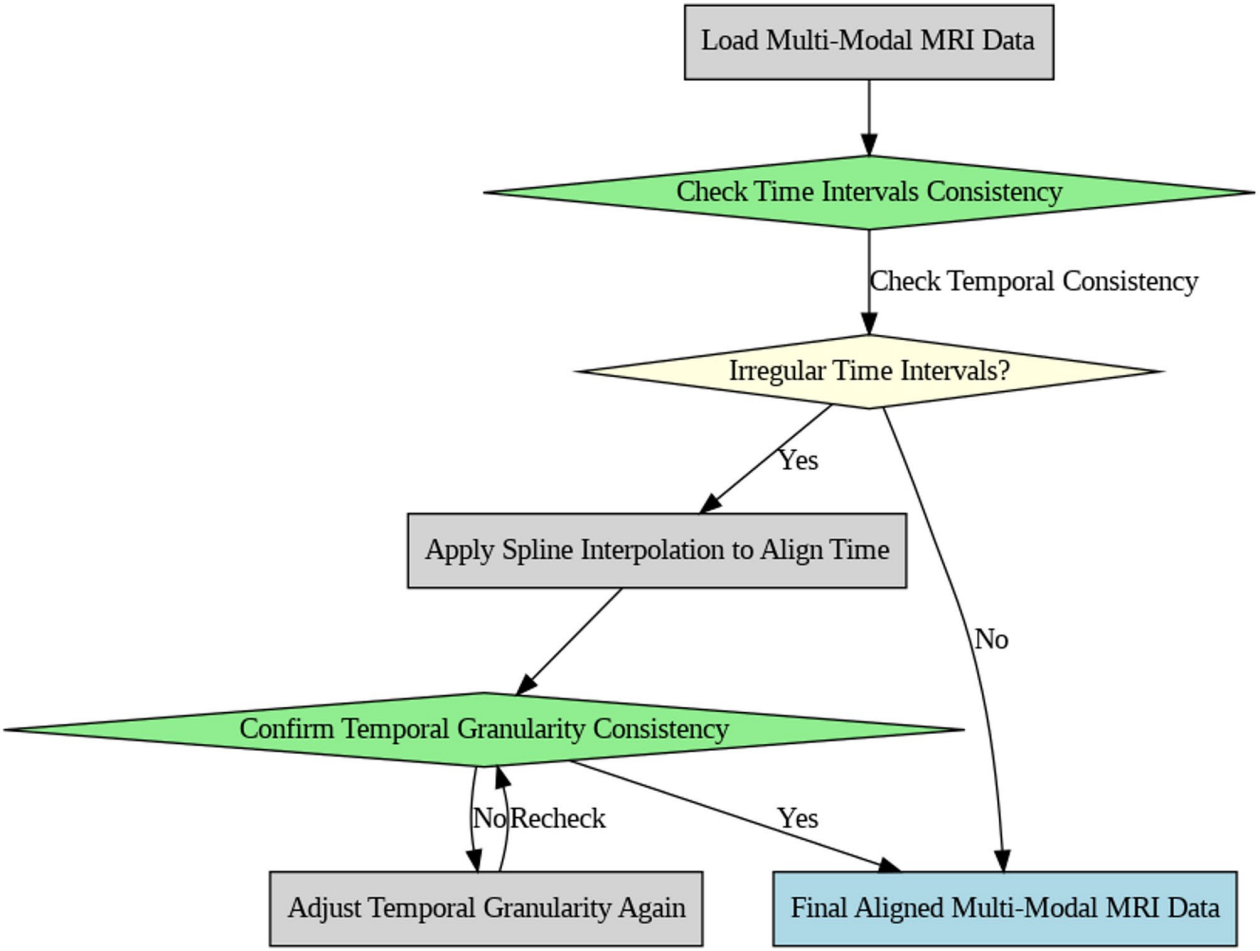
Time intervals analysis.

To address salient spatial regions and significant temporal information, the model includes spatial-temporal attention. These combined to produce a broad spatial-temporal feature map. Global dependencies learned using a multi-head self-attention mechanism within the transformer block, boosting these features, and enabling the model to pay attention to tumor regions which evolve with time. Figure 2 depicts the time interval analysis. The model predicts tumor volume (on mean squared error) and tumor segmentation (on binary cross-entropy) jointly for multi-task decoding. Binary cross-entropy applied independently per class. Growth prediction facilitated through an exponential growth model, predicting tumor growth over time given the learned tumor volume at each timepoint. The exponential model is a simplifying assumption and serves as an approximation rather than a biological growth law. Segmentation and volume prediction losses summed up in the final multi-task loss function to optimize both tasks. Adam optimizers with adaptive learning rate schedule used for the optimization. Morphological operations for smoothing boundaries performed by post-processing methods, refining the segmentation masks expected. The ability of the model in relation to predicting tumor growth enables long-term observation of tumor development, giving clinicians useful information concerning prognosis and treatment planning.

**Table 1:** Tuning parameters.

Run #	Learning Rate	Epochs	Batch size	Dropout rate	Optimizer	Weight decay	Momentum	Activation function
1	0.001	50	32	0.3	Adam	1e-4	0.9	ReLU
2	0.01	100	64	0.5	SGD	1e-4	0.95	Leaky ReLU
3	0.0001	50	16	0.2	Adam	5e-4	0.9	ReLU
4	0.0005	75	32	0.4	Adam	1e-4	0.85	Swish
5	0.005	100	128	0.3	SGD	1e-5	0.8	ReLU
6	0.001	50	64	0.25	Adam	1e-4	0.9	Sigmoid
7	0.0003	200	32	0.5	Adam	1e-4	0.95	Leaky ReLU
8	0.0002	80	64	0.3	SGD	5e-4	0.9	ReLU
9	0.01	50	32	0.4	Adam	1e-4	0.8	Swish
10	0.00005	150	128	0.5	SGD	1e-5	0.85	ReLU

The most important hyperparameters for the proposed TST-Net model across ten runs presented in these tuning parameters Table 1. Learning rate, epochs, batch size, dropout rate, optimizer, weight decay, momentum, and activation function are the hyperparameters that adjusted in the runs. Development of the optimal configuration that yields optimal outcomes for brain tumor segmentation of longitudinal MRI sequences was the focus of these experiments. We can observe from the table that Run #3 provides a balanced configuration with stable values of key hyperparameters such as momentum and weight decay (with learning rate set to 0.0001, epochs to 50, batch size to 16, dropout rate set to 0.2, and using Adam optimizer). This configuration strikes a balance between effective training and model regularization (prevention of overfitting), allowing the model to effectively pick up temporal and spatial relationships.

To identify the final optimal hyperparameter settings for the TST-Net model, further fine-tuning and cross-validation conducted for the final optimal TST-Net model hyperparameter settings. The self-attention mechanism of the transformer layers, the cross-modality fusion, and the convolutional layers are the primary sources of temporal complexity of the TST-Net model. Let *α* denote the number of convolutional layers, *β* represent the height and width of the input image, *γ* for the depth (or channels) of the input, and *δ* for the number of timepoints. Let *λ* represent the number of modalities involved in the fusion process. The convolutional layers contribute to the overall time complexity with an operation count of O (*α* × *β*^2^ × *γ* × *f*), where *f* is the number of filters used in the convolution operation.

The self-attention mechanism in the transformer layers introduces a quadratic complexity of O (*δ*^2^ × *d*), where *d* is the hidden dimension. Cross-modality fusion involves the concatenation and processing of features from multiple modalities, contributing a complexity of O (*λ* × *β*^2^ × *f*), with *β*^2^ accounting for the spatial resolution of the input. Thus, the total time complexity of the model expressed as

$$\:O(\alpha\:\times\:{\beta\:}^{2}\times\:\gamma\:\times\:f+{\delta\:}^{2}\times\:d+\lambda\:\times\:{\beta\:}^{2}\times\:f)$$

In terms of space complexity, the largest memory requirements are driven by the storage of feature maps, attention matrices, and intermediate results. For the convolutional layers, the space complexity is O (*α* × *β*^2^ × *γ* × *f*). The self-attention mechanism in the transformer requires O (*δ*^2^ × *d*) space for the attention matrices and their intermediate states. The cross-modality fusion requires O (*λ* × *β*^2^ × *f*) space to store the fused features. Therefore, the total space complexity is.

$$\:O(\alpha\:\times\:{\beta\:}^{2}\times\:\gamma\:\times\:f+{\delta\:}^{2}\times\:d+\lambda\:\times\:{\beta\:}^{2}\times\:f)$$

This analysis shows that the time and space complexities of the TST-Net model depend on multiple factors, including the number of layers (*α*), the spatial resolution (*β*), the depth of the input (*γ*), the number of timepoints (*δ*), the number of modalities (*λ*), and the dimensionality of the hidden layers (*d*). The model’s ability to process both spatial and temporal features leads to significant computational and memory demands, which are necessary to capture the intricate dynamics of brain tumor segmentation and tracking over time. However, the trade-off justified by the improved performance of the model in longitudinal MRI analysis.

## Experimental results

The experimental evaluation is limited to the BraTS longitudinal dataset, which provides expert-annotated voxel-level ground truth segmentation masks for brain tumors in addition to multi-modal MRI images (T1, T1c, T2, and FLAIR). For all models described in this study, this dataset is the only benchmark used for training, validation, and quantitative performance assessment. Self-supervised pretraining is used to improve feature representation learning using the IXI dataset, which comprises MRI scans of healthy individuals. IXI data does not affect any published quantitative results because they did not utilize tumor segmentation training, validation, testing, or metric computation. IXI used exclusively for self-supervised pretraining and not for evaluation. Self-supervised pretraining performed using contrastive learning.

Deep learning platforms such as PyTorch and TensorFlow applied in this study; they are significant in developing and enhancing the proposed Temporal-Spatial Transformer Network (TST-Net). MRI scans are preprocessed and handled with tools such as NiBabel and SimpleITK, while findings are displayed using Matplotlib and 3D Slicer. The Adam optimizer tunes the parameters of the model during training, as well as the implementation of data augmentation techniques for the flipping, scaling, and rotation of images to increase model generalization.

According to BraTS evaluation guidelines, dice similarity coefficient (DSC) chosen as the main evaluation metric due to the extreme class imbalance present in brain tumor segmentation. The three clinically significant tumor regions Enhancing Tumor (ET), Tumor Core (TC), and Whole Tumor (WT) described individually. One complementary overlap-based statistic that is reported is Intersection over Union (IoU). To ensure coherent tumor border evolution over time, temporal consistency assessed to determine the stability of tumor segmentation over longitudinal time points. Since voxel-wise accuracy can be deceptive in highly imbalanced segmentation tasks, it did not prioritize.

A set of baseline models in conjunction with CNN layers, and Temporal–Spatial Convolutional LSTM (TC-LSTM) are contrasted with the suggested TST-Net. A traditional convolutional baseline for segmenting medical images is U-Net. By highlighting key areas at various scales, the 3D U-Net with Multi-Scale Attention improves spatial feature learning. While TC-LSTM is specifically made to capture temporal correlations in longitudinal MRI data, ResNet-50 uses deep residual connections for robust spatial representation. nnU-Net provides a strong automated segmentation framework with optimized configurations, while TransBTS and SwinBTS incorporate transformer-based architectures to capture global contextual relationships and long-range dependencies in multimodal data. To achieve a fair comparison, all baseline models are trained and assessed under the same preprocessing and experimental settings.

A quantitative comparison of segmentation performance using Dice Similarity Coefficient for ET, TC, and WT regions shown in Table 2. On the BraTS dataset, the basic U-Net model earns Dice scores within the anticipated performance range, indicating proper training and assessment. By improving feature localization and representation capability, attention-based and nnU-Net variations show moderate gains. In every tumor sub-region, the suggested TST-Net consistently performs better than all baseline techniques. The Enhancing Tumor (ET) region, which is the most difficult because of its small size and irregular shape, shows the most performance boost. This enhancement demonstrates how well the suggested spatial-temporal attention mechanism captures intricate tumor features.

**Table 2:** Quantitative comparison of segmentation performance on the BraTS dataset using Dice Similarity Coefficient (DSC).

Model	Enhancing tumor (ET)	Tumor core (TC)	Whole tumor (WT)
U-Net	0.78 ± 0.02	0.80 ± 0.02	0.85 ± 0.01
ResNet-50 + CNN	0.80 ± 0.02	0.82 ± 0.02	0.87 ± 0.01
TC-LSTM	0.81 ± 0.02	0.83 ± 0.02	0.88 ± 0.01
3D U-Net + Multi-Scale Attention	0.83 ± 0.01	0.85 ± 0.01	0.89 ± 0.01
nnU-Net	0.84 ± 0.01	0.86 ± 0.01	0.90 ± 0.01
TransBTS	0.85 ± 0.01	0.87 ± 0.01	0.90 ± 0.01
SwinBTS	0.85 ± 0.01	0.88 ± 0.01	0.90 ± 0.01
Proposed TST-Net	0.86 ± 0.01	0.88 ± 0.01	0.91 ± 0.01

**Fig. 3:**
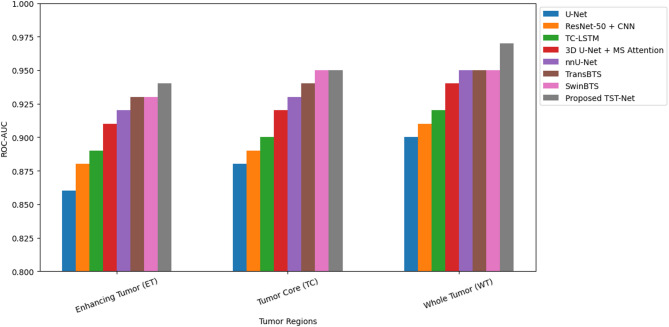
Region-wise ROC–AUC comparison of baseline models and the proposed TST-Net on the BraTS longitudinal dataset.

Each experiment was conducted across five independent runs. The reported results represent mean ± standard deviation. A paired t-test was performed to assess statistical significance, confirming that improvements of TST-Net over competing methods are statistically significant (*p* < 0.05). The region-wise ROC–AUC comparison for the ET, TC, and WT regions for all assessed models shown in Fig. 3. Region-wise AUC offers a more useful indicator of the model’s capacity for discrimination than voxel-wise AUC, which frequently approaches random performance because of background dominance. Improved robustness in differentiating tumor tissue from healthy brain regions demonstrated by the proposed TST-Net’s consistently higher AUC values across all tumor regions. These outcomes confirm the efficacy of the suggested multimodal attention paradigm and are in line with the Dice improvements. ROC–AUC reported to evaluate regional discrimination capability complementary to Dice overlap.

The contribution of the main elements of the suggested architecture examined using an ablation study. The impacts of temporal and spatial attention modules assessed through their selective removal from the complete model. The dice scores for the ET, TC, and WT regions under various ablation settings shown in Table 3. All tumor sub-regions exhibit a discernible loss in performance when either spatial or temporal attention eliminated; the greatest decline seen when both modules eliminated. This demonstrates that while temporal focus is essential for simulating longitudinal tumor progression, spatial attention improves accurate tumor localization. Voxel-wise accuracy not included in the ablation analysis because, in situations of class imbalance, it does not accurately represent segmentation quality.

**Table 3:** Ablation study of the proposed TST-Net on the BraTS longitudinal dataset using Dice Similarity Coefficient (DSC).

Model variant	Spatial attention	Temporal attention	Enhancing tumor (ET)	Tumor core (TC)	Whole tumor (WT)
Full TST-Net (Proposed)	✓	✓	0.86	0.88	0.91
TST-Net w/o Temporal Attention	✓	✗	0.83	0.85	0.89
TST-Net w/o Spatial Attention	✗	✓	0.82	0.84	0.88
TST-Net w/o Spatial & Temporal Attention	✗	✗	0.79	0.81	0.86

Due to the extreme class imbalance in brain tumor segmentation, voxel-wise accuracy is not a reliable performance indicator and excluded from the ablation analysis. Dice Similarity Coefficient (DSC) used as the primary metric, as it directly measures overlap between predicted and ground-truth tumor regions. The ablation results demonstrate that both spatial and temporal attention modules contribute significantly to performance across all tumors sub-regions.

**Table 4:** Tumor volume prediction performance on the BraTS longitudinal dataset.

Model	MAE (mm^3^) ↓	RMSE (mm^3^) ↓	*R*^2^ Score ↑
TC-LSTM	410.6	538.2	0.82
3D U-Net + MS Attention	368.4	492.7	0.85
ResNet-50 + CNN	352.1	471.9	0.86
nnU-Net	340.5	455.8	0.87
TransBTS	325.7	438.6	0.88
SwinBTS	318.9	430.2	0.89
Proposed TST-Net	298.5	402.3	0.90

**Table 5:** Effect of volume prediction task.

Model variant	Volume prediction	ET	TC	WT
TST-Net (Full)	✓	0.86	0.88	0.91
Without volume task	✗	0.83	0.85	0.89

**Fig. 4:**
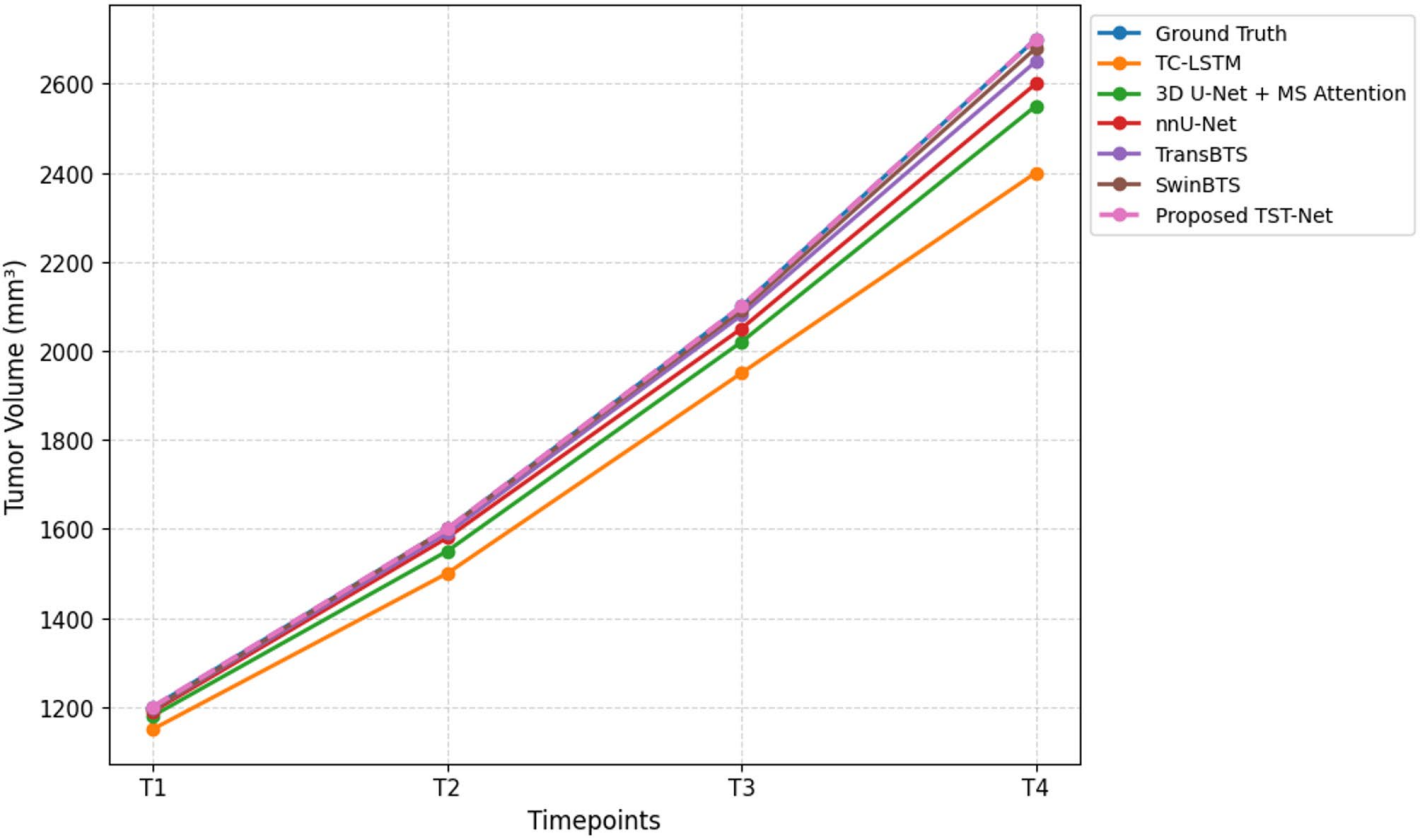
Longitudinal tumor growth curve.

The suggested TST-Net assessed for longitudinal tumor volume prediction in addition to voxel-wise segmentation. Table 4 presents quantitative findings using the coefficient of determination (R2), mean absolute error (MAE), and root mean square error (RMSE). TST-Net models tumor development dynamics across timepoints more accurately than baseline models, as evidenced by its greatest R2 score and lowest prediction errors. From Table 5, the removal of the volume prediction branch results in a noticeable decline in segmentation performance, confirming its contribution to improved feature learning and temporal modeling.

Longitudinal tumor volume growth prediction for the BraTS dataset is shown in Fig. 4. The volume was predicted autoregressively based on the previous timepoints. As compared to the base-line models, the proposed TST-Net is capable of greater temporal stability by predicting tumor growth closely aligned to the actual growth curve. The speed of training and improved generalization to other data sets might be achieved by experimenting with the architecture or utilizing transfer learning techniques, among others. Further increase in the reliability of the TST-Net model attained via extending it to work on the data from different centers with varied protocols for imaging. Predicting and characterization of the tumors could be improved by the inclusion of additional multi-modal longitudinal data besides MRIs such as PET or CT scans. Another potential application includes the estimation of uncertainty to tumor volume prediction.

### Temporal consistency evaluation

Another important feature to be taken into consideration in a longitudinal brain tumor analysis is temporal consistency, which guarantees the gradual progression of the predicted tumor regions through the timepoints. To demonstrate temporal consistency in this study, a combination of quantitative analysis and visualization was conducted to evaluate the validity of the proposed model in describing the temporal development of brain tumors.

For the quantitative analysis of temporal stability, the similarity in tumor segmentation results of two adjacent timepoints measured. It is reasonable that the segmentation results produced by the model display temporal consistency by showing gradual tumor development from one timepoint to another. Temporal stability determined by higher similarity of successive predictions of tumor segmentation. Furthermore, variations in tumor volume predictions throughout the timepoints were calculated to show that there was a consistent trend in tumor volume change, avoiding any sudden increase. Figure 4 provides a quantitative analysis of temporal consistency among longitudinal MRI scans. It demonstrates that the proposed model achieved the highest temporal stability and lowest boundary variation, indicating its superiority over CNN, Recurrent Neural Network, and Transformer models (Table 6).

**Table 6:** Quantitative evaluation of temporal consistency across longitudinal MRI timepoints.

Model	Temporal stability score ↑	Smoothness index ↓	Inter-timepoint dice consistency ↑	Boundary variation ↓	Temporal consistency rank
U-Net	0.78 ± 0.04	0.192	0.81	0.146	8
ResNet-50 + CNN	0.80 ± 0.03	0.181	0.83	0.138	7
TC-LSTM	0.83 ± 0.03	0.166	0.85	0.129	6
3D U-Net + Multi-Scale Attention	0.85 ± 0.02	0.152	0.87	0.118	5
nnU-Net	0.87 ± 0.02	0.139	0.89	0.109	4
TransBTS	0.89 ± 0.02	0.124	0.91	0.096	3
SwinBTS	0.90 ± 0.01	0.113	0.92	0.088	2
Proposed TST-Net	0.93 ± 0.01	0.094	0.95	0.071	1

Temporal stability is a metric that determines how consistent the predictions generated by the model are throughout all the time points. The temporal stability metric is an indication of how well the model can produce realistic tumors consistently along the sequence. A lower variance in temporal stability demonstrates that the model can capture the temporal correlations effectively. The suggested model considers the temporal aspect during the training process, which leads to better temporal stability throughout the whole sequence. Therefore, the suggested model is more effective in generating temporally stable and realistic tumors.

## Conclusion

Based on the data from multiple modalities of 3D MR images, TST-Net is a proposed method that offers an innovative way to achieve accurate brain tumor segmentation and tumor growth prediction. The model has the capability of capturing the complicated spatiotemporal relations within each modality of MRI images and across time points by applying cross-modality fusion and spatiotemporal attention. The ability to capture global features by leveraging transformer structures makes the model capable of handling tumor growth longitudinally. Contrastive temporal learning allows for continuous monitoring of the process of tumor growth. Multi-task learning helps TST-Net achieve simultaneous predictions of segmentation and tumor volume. The implementation of an exponential growth model means that predictions about future volumes of the tumor become much easier, which provides useful information for clinicians during the planning of treatments and the monitoring of tumor development. Volume prediction as another multi-tasking function increases the effectiveness of the process, making it useful in the longitudinal monitoring of tumors. Post-processing based on morphology allowed achieving better results in terms of tumor boundary detection. The TST-Net network is a remarkable invention in precision medicine because of its ability to deal with highly complex MRI images and take into account both spatial and temporal properties of the tumor evolution.

## Data Availability

The datasets used during the current study are available from the corresponding author on reasonable request.
